# Blood Platelets as an Important but Underrated Circulating Source of TGFβ

**DOI:** 10.3390/ijms22094492

**Published:** 2021-04-26

**Authors:** Kamil Karolczak, Cezary Watala

**Affiliations:** Department of Haemostatic Disorders, Medical University of Lodz, ul. Mazowiecka 6/8, 92-215 Lodz, Poland; cezary.watala@umed.lodz.pl

**Keywords:** TGFβ, platelets, Smad, cardiovascular, granules, secretion, fibrosis, preeclampsia

## Abstract

When treating diseases related primarily to tissue remodeling and fibrosis, it is desirable to regulate TGFβ concentration and modulate its biological effects. The highest cellular concentrations of TGFβ are found in platelets, with about 40% of all TGFβ found in peripheral blood plasma being secreted by them. Therefore, an understanding of the mechanisms of TGFβ secretion from platelets may be of key importance for medicine. Unfortunately, despite the finding that platelets are an important regulator of TGFβ levels, little research has been carried out into the development of platelet-directed therapies that might modulate the TGFβ-dependent processes. Nevertheless, there are some very encouraging reports suggesting that platelet TGFβ may be specifically involved in cardiovascular diseases, liver fibrosis, tumour metastasis, cerebral malaria and in the regulation of inflammatory cell functions. The purpose of this review is to briefly summarize these few, extremely encouraging reports to indicate the state of current knowledge in this topic. It also attempts to better characterize the influence of TGFβ on platelet activation and reactivity, and its shaping of the roles of blood platelets in haemostasis and thrombosis.

## 1. Introduction

Since discovering that platelets contain and secrete platelet growth factor (PDGF), there has been a significant growth in interest in these cells, arising as anucleated fragments of the megakaryocyte cytoplasm, as the carriers of growth factors [1]. Of these growth factors, transforming growth factor beta (TGFβ), a protein secreted in huge amounts by platelets, is of particular interest. As TGFβ exerts a considerable range of pleiotropic molecular effects on cell physiology [2,3,4,5], there is a need gather current knowledge on the role of platelets as circulating carriers of TGFβ in an organism.

This paper is based on a review of articles published since 1974 through 2020, found in the Medline database using keywords “*TGFb*” [AND] “*blood platelets*”. Out of 98 references cited in the article, 45 papers come from the period of 2010–2020, 38 from the period of 2000–2009, nine from the years 1990–1999, five between 1980–1989 and one citation appeared in the 1970s. It is evident that interest in platelet TGFβ has intensified in the last two decades, which makes the subject quite a new one in Medicine. Obviously, this implies the existence of a vast number of unanswered questions in the area, despite its strong clinical relevance. Therefore, the aim of this paper is to review the current state of knowledge regarding the activity of TGFβ secreted by blood platelets; given the considerable breadth of the topic, our discussion is restricted to the available evidence concerning diseases documented to be associated with platelet-derived TGFβ.

## 2. TGFβ in Blood Platelets

It is widely believed that platelets are the leading carrier of TGFβ in the body, containing 40 to 100 times more TGFβ than other cells [1], with 45% of the basal plasma TGFβ level believed to derive from platelets [6,7]. However, this statement is quite an “old truth”, and it would be interesting to revisit this ranking using more modern approaches.

Even so, several studies have shown a positive correlation between the number of platelets and the concentration of TGFβ in the peripheral blood [8,9,10,11,12], confirming that they play an important role as active carriers of TGFβ. However, it is important to note that the literature regarding this role is not unequivocal [13].

Early results revealed that TGFβ is stored in platelets as an inactive high molecular weight complex that becomes activated when secreted into the extracellular environment [14,15]. This complex contains the activable form of TGFβ and a masking glycoprotein. The masking protein is composed of one large subunit of about 110 kDa and two small subunits, each of 39 kDa, linked by disulphide bridges.

However, it is important to emphasize that this is not a structural feature of TGFβ unique to platelets. Most cells store and secrete TGFβ in the form of complexes containing either the latency-associated peptide (LAP) or with the latent TGFβ binding protein (LTBP) [16]. Occasionally, TGFβ complexes may also be formed with other proteins, such as the latent TGF-beta complexed protein-1 (LTCP-1) [16]. It would be clearly advantageous for future studies to examine the formation of TGFβ complexes with proteins other than LAP and LTBP in platelets.

Blood platelets also contain the complexed TGFβ form, most commonly TGFβ1, with the other TGFβ isoforms contributing only about 5% in total [17]. Due to this predominance, the term TGFβ in this paper will refer mainly to TGFβ1, since other subtypes in blood platelets can simply be neglected.

TGFβ is present in two separate pools, which are stored in the alpha granules. The first pool, representing 95% of the total TGFβ present in platelets, comprises molecules of TGFβ (the mature TGFβ, MW 25 kDa) complexed with LTBP and LAP. The second pool comprises the two-component complex composed of TGFβ and LAP, without LTBP. Thus, two structurally-different complexes of TGFβ exist in blood platelets.

The TGFβ molecules can be secreted into blood plasma in two distinct ways. Briefly, the first pool of platelet TGFβ (TGFβ+LTBP+LAP) is released into plasma during the process of blood clotting as a one-step reaction; however, in the case of the second pool (TGFβ+LAP), the protein is not released directly into plasma—it becomes “trapped” in the clot before being later released into plasma after activation by RGD peptide.

The physiological significance of this double-mode secretion of TGFβ from platelets remains unexplored, but its role in the process of wound healing has been suggested [17]. The possible importance of biphasic TGFβ secretion in the aetiology of diseases has not yet been considered. Hence, its importance in the development of cardiovascular diseases, tumour metastasis, liver disease, cerebral malaria, or tissue regeneration remains unknown.

Platelets secrete latent TGFβ and activate it shortly after release. This process also occurs after removal of the platelets, suggesting that this activation process depends on the presence of a factor secreted from platelet granules together with TGFβ; however, while activation may not necessarily require the direct presence of intact platelets, it is certainly dependent on the compounds present in platelet releasate. This activating factor appears to be an enzyme similar to furin-like proprotein convertase; as such, the activation of TGFβ by platelet secretome may be not be dependent on the classical TGFβ activators known from other cell types [18]. Therefore, the factor(s) secreted from the platelet granules together with TGFβ are necessary for its full activation. In the absence of these activating factors, full activation of TGFβ may not occur; this can be seen in the case of type I collagen synthesis by fibroblasts, which is only initiated by the action of TGFβ and is greatly increased in the presence of substances secreted by platelets. The nature of these substances, however, remains to be determined [9].

However, TGFβ is understood to be secreted from platelets upon the action of agonists. Some of these compounds have been identified so far, with the best recognized being thrombin and collagen, with no references, however, to ADP and arachidonic acid. The molecules have differing effects: While thrombin generates a cytokine “burst”, collagen stimulates what appears to be a prolonged leakage of TGFβ from platelets [19].

The concentration of TGFβ secreted by platelets is critical to maintain the clinically important properties of platelet-rich plasma, which is currently being investigated for numerous therapeutic applications, which are discussed below. Hence, it is valuable to understand the kinetics and the mechanism(s) of TGFβ secretion in a “single ejection” or a “prolonged leakage” under the influence of various platelet agonists. Unfortunately, no complete comparison of all known platelet agonists has yet been carried out in terms of their ability to stimulate TGFβ secretion from platelets.

Less standard platelet agonists (or co-agonists), like some chemokines, are also able to stimulate TGFβ release from blood platelets. For example, the stromal cell-derived factor 1 (SDF-1, the C-X-C Motif Chemikine Ligand 12 [CXCL12]) can stimulate release via stimulation of the alpha-chemikine receptors specific for SDF-1, CXCR4 and CXCR7. An association has been found between the concentration of TGFβ bound to platelet membranes, the concentration of SDF-1 on the platelet surface and the membrane expression of CXCR4 and CXCR7 receptors [20]. It is important to note that all the diseases discussed below, i.e., ones influenced by platelet TGFβ, are characterized by changes in the synthesis of SDF-1 [21,22,23,24,25]; it can be seen that the pathway leading in blood platelets from SDF-1 receptors to TGFβ secretions requires careful exploration.

Interestingly, neither age nor gender have been claimed so far to affect the levels of TGFβ produced by platelets, this evidence however should be re-evaluated and confirmed in larger study groups [8].

## 3. TGFβ Regulates Platelet Reactivity

Platelets are not only able to secrete TGFβ and to induce TGFβ-dependent responses in other target cells, but they also represent a cellular target for TGFβ and are TGFβ-sensitive themselves [26]. This is possible because blood platelets express the TGFβ type II receptor, which significantly affects their basic functions. At lower ADP concentrations, increased platelet aggregation was observed, regardless of the exposure time to TGFβ. Similar increase in platelet reactivity was observed at higher ADP concentrations, but only after a longer incubation with TGFβ. In contrast, platelet reactivity decreased when a higher concentration of the agonist was applied and the platelet sample was exposed to TGFβ for a shorter time. The authors attribute these seemingly opposite effects to the bimodal impact of TGFβ on platelet reactivity. However, the general picture arising from these results is that TGFβ enhances platelet reactivity to ADP, albeit with a singular exception. In this experiment, it is possible that the longer period of blood platelet incubation with TGFβ (one hour) resulted in significant in vitro platelet consumption, which could be particularly likely in the case of platelet-rich plasma. As a result, it is possible that some artefactual fluctuations in platelet reactivity might have appeared. Nevertheless, these results obtained in vitro may have important in vivo implications: under circumstances when concentration of TGFβ is increased in blood and platelets locally increase ADP concentration, as a result of activation and secretion of ADP from their granules, the autocrine activatory action of ADP on platelets may again be increased, thus further augmenting thrombosis. This mechanism is especially probable in thrombosis associated with myeloproliferative disorders [26].

Platelets have also been shown to contain the Smad2 protein, which becomes phosphorylated in response to the action of TGFβ; thus, the Smad2 protein can be certainly proposed as a pivotal transmitter of TGFβ signal in platelets [26].

The fact that the Smad2 protein and its phosphorylation plays a role in stimulation by platelet TGFβ opens a wide field of future research on Smad2 activation as a useful marker of in vivo platelet responsiveness to TGFβ. However, the findings of simple studies correlating platelet activation and reactivity with plasma TGFβ levels may appear to be insufficient, or even confusing, since the final direction of platelet responsiveness of blood platelets will depend not only on TGFβ concentration, but also on local concentrations of platelet agonists. Hence, further studies are needed on the relationships between blood platelet TGFβ and the phosphorylation of the effector protein, Smad; this may well serve as a more useful marker of platelet sensitivity to TGFβ than a combination of TGFβ blood concentration and markers of platelet functional state, such as aggregability or expression of membrane markers of activation. A number of studies have examined the role of Smad family proteins in transmitting cell signals from TGFβ receptors, and they are now widely recognized as key mediators in the cell response to TGFβ stimulation [27,28,29]. However, in contrast to studies in nucleated cells, research on the roles of TGFβ receptors and Smad proteins in platelets remain largely uncommon and at an early stage. Even so, it should be remembered that the functional effects of physiological and artificial modulators of TGFβ receptors and their effector proteins may have a significant role in the regulation of primary haemostasis, and thus for the treatment of thromboembolic diseases. These diseases, which can lead to serious thrombotic events, appear to be highly influenced by TGFβ.

It should also be emphasized that the signal transmitted from TGFβ receptors by Smad proteins terminates in the cell nucleus, where Smad proteins accumulate and regulate the process of gene transcription. There is a need for further studies examining the molecular mechanisms of action of TGFβ and its effector proteins in anucleated platelets; their physiology is already known to be significantly regulated by the same proteins that act as transcription factors in nucleated cells [30].

Although little is known about the exact mechanism by which TGFβ is secreted from the platelet granules, it appears to be a highly-regulated process. For example, the levels of TGFβ in the peripheral blood of patients with systemic sclerosis do not differ significantly from those found in that of control volunteers. This suggests that TGFβ should not be associated with disorders characteristic of systemic sclerosis, such as angiogenesis disorders: these may be more associated with Vascular Endothelium Growth Factor (VEGF), which is present at higher levels in patients with systemic sclerosis. This rise in VEGF blood levels is believed to be largely due to the blood platelets demonstrating greater activation and secreting functional VEGF at higher concentrations than in healthy people. This augmented release can be blocked by iloprost [31].

These findings on VEGF and TGFβ in systemic sclerosis indicate that the general secretion of TGFβ or growth factors from blood platelet granules is a specific and selectively regulated process. It is impossible certainly to say that each type of protein present in platelet granules is secreted in the same way, i.e., a simple gradual discharge of substances from the cell, like from a tear in a bag. The process bears specific regulatory features, probably unique to each of the proteins enclosed in platelet granules, which remain little understood.

## 4. The Platelet Membrane-Bound TGFβ Pool and Its Possible Involvement in a Modulation of Platelet Reactivity and Platelet-Leukocyte Interactions

TGFβ is believed to exist in two pools in blood platelets. These differ not only according to its complexation with additional proteins, mention above, but also according to subcellular intraplatelet compartmentalization. TGFβ is present not only inside the platelet cytoplasm, i.e., in alpha granules, but also on the outer surface of the outer platelet membrane, where it is attached via glycoprotein A repetitions predominant protein (GARP). The presence of the TGFβ-anchoring GARP protein on the platelet surface is quite a unique feature, only being detected elsewhere on FOXP3+ regulatory T cells; i.e., not on any other tested cell, including CD14 monocytes, CD8 T cells, natural killer cells, NK T-cells or CD19/CD20B cells [32].

More detailed studies are needed to determine the conditions that influence GARP expression on the surface of platelets, as well as other cell types of the immune system, or rather, of the thromboimmune system. Furthermore, due to their exceptionally high concentrations of TGFβ and the unique presence of GARP, platelets should be included in any studies on the role of GARP in TGFβ binding [32].

If we assume that TGFβ released from platelets can interact with the forkhead box P3 protein (FOXP3, also known as scurfin)-positive (FOXP3+) regulatory T cells, the other cells that also express GARP, it can be further hypothesized that platelets may somehow regulate the mechanism of infectious tolerance and the immune response of FOXP3+ cells to antigens exposed on antigen-presenting cells (APCs), converting FOXP3- T cells into FOXP3+ counterparts. Similarly, it has also been suggested that platelet GARP may play a role in regulating other key TGFβ-dependent responses, such as the uptake of TGFβ by cancer cells, leading to a weakened response of the immune system [32].

A number of biological processes are highly dependent on TGFβ, such as tissue fibrosis, tissue remodeling and inflammation; however, the extent to which they are regulated by platelet GARP, probably by binding or inactivating TGFβ to the platelet and releasing it into circulation, remains poorly understood. It also remains unclear whether changes in GARP protein expression are involved only in the regulation of the plasma membrane-bound TGFβ pool, or whether it can also regulate the intracellular TGFβ pool, thus influencing its secretion from the cells.

Platelets are not the only blood cells capable of secreting TGFβ; monocytes and neutrophils exhibit a similar property [33]. TGFβ derived from neutrophils and macrophages is an active factor and produces its typical effects in target cells, two key ones being the epithelial to mesenchymal transition in bronchial epithelial cells [34] or the production of amphiregulin by intestinal epithelial cells [35]. Considering that platelets express the TGFβ receptor and that TGFβ is a modulator of platelet ADP reactivity [26], it can be expected that immune cell-derived TGFβ may be involved in the cross-talk of platelets with monocytes and neutrophils. We already know that these cells interact with each other and that their mutual interactions are of considerable physiological importance. It is important to note the ability of platelets to stimulate the transition of monocytes to macrophages [36], to enhance the adhesion of monocytes complexed with platelets to the endothelium [37], and for platelets opsonized with IgG antibodies to transform monocytes into cells that secrete Il-10, an important anti-inflammatory cytokine) [38]. Platelets are also known to stimulate the oxidative burst, form cell traps and stimulate phagocytosis in neutrophils [36]. Thus, we already know that platelets interact directly with inflammatory cells, both in laboratory conditions and in living organisms. Clearly, more research is needed regarding the contribution of TGFβ to these interactions, particularly since platelet TGFβ is known to be of crucial importance in the regulation of the immune system: platelet TGFβ enhances the antigen-specific suppressor response by converting conventional T (T_conv_) lymphocytes to functional regulatory lymphocytes (T_reg_) [39].

In addition, platelet TGFβ has been found to ameliorate the cytotoxic properties of NK cells in women with endometriosis, reflected in decreased NKG2D expression in NK cells induced by platelet TGFβ. Interestingly, in women with endometriosis, the concentration of TGFβ in the peritoneal fluid correlates with the extent of blood platelet activation [40].

Thus, the interactions between platelets and immunocompetent cells are of physiological and clinical importance and should be studied more intensively in the further future.

## 5. Measurements of Platelet-Derived TGFβ: Some Methodological Considerations

Platelets act as cellular reserves of TGFβ, and can release it quickly into the circulation during the activation process. These cells hence play an important role in governing the concentrations of TGFβ in the peripheral blood. This feature has very important methodological implications. For example, for reliable measurements of intraplatelet TGFβ concentrations, or assessments of TGFβ secretion from platelet granules, special precautions need to be taken to avoid artefactual activation of the platelets when collecting blood, isolating platelet-rich plasma or preparing platelet suspensions in an artificial buffer.

The concentration ranges of TGFβ in humans and animals are currently described as extremely wide, both under pathological and physiological conditions. In order to obtain reliable results when assessing the concentration of TGFβ, blood samples should be fractionated quickly and smoothly; any delay in processing could increase the risk that elevations in TGFβ concentrations may be due to artefactual activation of platelets rather than impairments occurring in the monitored organism. To prevent such artefactual platelet activation, it is advisable to use factors such as prostaglandin E1 [6].

The greatest risk factor for artefactual stimulation of platelets is exposure to excessive shear forces, which can obviously happen at any stage during the processing of a blood sample. Briefly, during blood collection, platelets may experience shear forces when blood flows through the aspirating needle or if it is sucked into the tube under too high pressure. It can also occur during sample centrifugation at excessive g-forces. Likewise, slowing the centrifugal rotor too quickly may also cause a shear stress, resulting in the “loss” of a significant pool of TGFβ from platelets. Therefore, only the most gentle and sparing methods should be used when isolating blood platelets [41,42].

Two key points should be considered when obtaining reliable blood TGFβ results. Firstly, blood serum typically yields higher TGFβ values than plasma. Most of the TGFβ measured in the serum sample is released from platelet granules during the clotting-associated activation preceding serum isolation. Moreover, it should be remembered that most of the TGFβ in the circulating blood is complexed with proteins, e.g., α_2_-macroglobulin; the presence of these complexes can mask the signal of TGFβ and result in dilutional nonlinearity. Therefore, any measurement should employ an the appropriate method to reduce the re-association effect between TGFβ and complexing proteins [43].

These points are of particular importance for research on platelet-rich plasma (PRP) as a factor stimulating healing and regeneration. TGFβ is essential for the wound healing and tissue regeneration process [44,45], and its process largely determines the regenerative properties of PRP. Inadequate isolation may lead to the recovery of PRP with low TGFβ levels and thus low regeneration efficiency. In addition, accurate estimation of this efficiency requires a thorough knowledge of the basic physico-chemical properties of TGFβ in the environment of the obtained PRP sample.

## 6. Platelet TGFβ as a Key Component of Platelet-Rich Plasma Used in Regenerative Medicine

Autologous platelets, and more precisely their granular content, can be successfully used in regenerative medicine. In this case the particular attention should be paid to the preparation of platelet-rich plasma (PRP). An appropriate method of platelet concentration [46,47] will also concentrate TGFβ, increasing the healing abilities of the PRP; the resulting extract is of great use in oral maxillofacial surgery [46] or in veterinary procedures [47]. As plasma TGFβ concentrations naturally correlate with platelet count, PRPs with higher platelet concentrations will clearly have higher concentrations of TGFβ [47,48].

The TGFβ in the PRP accelerates the regeneration and significant recovery of intervertebral discs. The process, believed to be dependent on Smad proteins, has been associated with the increased expression of collagen II or aggrecan sox-9 and the decreased concentration of collagen X [49].

However, TGFβ is also able to dysregulate the mechanisms of homeostatic tissue repair, leading to enhanced fibrosis. Indeed, the presence of TGFβ in PRP has been associated with a highly-pronounced fibroproliferative effect stronger than bone matrix deposition, resulting in reduced deposition in the craniofacial unit [50]. Thus, platelet TGFβ has also the potential to impede normal bone regeneration.

While much is known about the role of TGFβ in wound healing and tissue regeneration, the results gathered so far only occasionally relate to platelets as a significant source of circulating TGFβ.

## 7. Platelet TGFβ in Cardiovascular Diseases

It is well known that TGFβ is involved in the development of the heart, as well as in vasculo- and angiogenesis [51]. Also, in adulthood the blood vasculature is one of the main sites of the action of TGFβ, exemplified in its involvement in cardiovascular diseases. In particular, patients with Marfan syndrome may have an increased risk of the aortic aneurism, proportional with the level of platelet TGFβ. However, higher levels of platelet aggregation induced by either ADP or collagen do not correlate with aortic diameter in Marfan syndrome [52]. Hence, there is a need for further, more reliable, evidence to determine the true role of platelet TGFβ in shaping the risk of aneurisms in Marfan syndrome.

In addition, myocardial hypertrophy and systolic dysfunction associated with pressure overload are two more examples of pathologies associated with tissue remodelling, in which the processes of fibrosis and calcification seem to be particularly dependent on platelet TGFβ [53]. Elegant evidence in this regard was provided by experiments based on silencing TGFβ gene expression in megakaryocytes; this was found to result in the silencing of TGFβ expression in blood platelets, which partially protects from the development of cardiac hypertrophy [6].

In patients with aortic valve stenosis, the shear-stress experienced by blood platelets is considered a very significant trigger for the progression of stenosis. However, existing data suggests that shear-stress, which indeed contributes to TGFβ release from blood platelets, may be not sufficient to induce aorta remodeling; in addition, some synergistic action of additional factors is needed to obtain full activation of blood platelets, the robust secretion of TGFβ from platelet granules and the response from the target TGFβ-sensitive cells of the aorta wall. Some interesting observations in this regard have been made on hypercholesterolemia. Very briefly, a significant correlation has been noted between the plasma TGFβ concentration in ApoE-/- mice fed a high-fat diet, and the levels of cholesterol, as well as with the levels of antibodies against oxidized LDL (oxLDL) [54]. Also, the TGFβ concentrations in blood plasma increased with the duration of the hypercholesteraemic diet [53].

Is it therefore possible that shear-stress may be the only, or decisive, factor in inducing the activation-associated release of TGFβ from platelets? The accumulated evidence suggests that the maximum concentration of TGFβ in circulating blood is associated with at most, only a moderate increase in shear forces, certainly not a high increase. Therefore, it should be recognized that the release of TGFβ from platelets is probably interwoven into the synergistic action of several factors, among which hypercholesterolemia might sensitise platelets to the action of shear forces generated in stenotic blood vessels. Elevated cholesterol levels can facilitate platelet activation and TGFβ release from platelets by even moderate shear forces, which would only slightly activate platelets in a normocholesterolemic environment. Clearly, the stimulation of TGFβ secretion associated with platelet activation requires the combined action of numerous factors, although shear-stress is still widely considered as one of the strongest stimuli contributing to this release, despite the fact that shear-stress alone may be too weak to induce a strong response. The co-activation induced by a factor like hypercholesterolemia may result in even moderate TGFβ secretion becoming maximal under special conditions. Hence, the significance of such a co-activation certainly should not be underestimated.

It seems likely that the local activation of platelets at the site of the formation of shear-stress leads to the release of TGFβ, which is quickly “consumed” by the cells of the vessel wall directly adjacent to activated platelets. TGFβ, locally secreted from platelets, activates intracellular signalling pathways in its vicinity, *inter alia* in valvular interstitial cells. The pathways induced by platelet TGFβ can be both canonical, i.e., dependent on Smad2/3 protein, or non-canonical, i.e., dependent on ERK1/2 protein. This suggests that TGFβ is not only secreted from platelets under shear-stress conditions, but it is also rapidly activated under these conditions and immediately trigger biochemical reactions in the target cells of various tissues including the vascular wall and heart.

Interestingly, the intracellular response to TGFβ does not appear to be dependent on the continuous supply of successive pools of TGFβ from activated platelets, but persists in target cells for longer periods of time, even when the inducers of TGFβ secretion from platelets, such as shear-stress or hypercholesterolemia, have long since disappeared from the body. Thus, the active form of TGFβ present in a circulating blood is in fact largely derived of platelets and it is difficult to be tracked in plasma with conventional immunochemical detection methods, mainly due to the rapid binding of TGFβ to target cells. Therefore, direct immunochemical detection of TGFβ in plasma is not sufficient for reliable prediction of responsiveness of cells to TGFβ, and activated Smad effector protein expression in target cells should be considered instead.

Therefore, shear-stress appears to play a significant role in allowing platelet TGFβ to influence other cells. It promotes the secretion of latent TGFβ from platelets into the extraplatelet environment; it also activates TGFβ and allows it to acquire the structural features necessary to affect the cytophysiology of target cells. As already mentioned before, the activation of platelet TGFβ is closely dependent on factors secreted simultaneously from platelet granules.

Thus, vasoconstriction induces shear-stress, which in turn, activates platelets. These cells further secrete TGFβ; when activated, this induces fibrosis and calcification of the vessel wall. As a result, the all narrows the vessel lumen even further, exacerbating local stenosis and increasing the local shear forces, resulting in a vicious cycle which further activates circulating platelets [53]. It is important not to neglect in this pathogenic cycle co-activation by additional atherogenic factors, like hypercholesterolemia.

Nevertheless, platelets are not widely recognized as being involved in the pathological or physiological processes induced by TGFβ in cardiovascular system. In an otherwise excellent review of the role of TGFβ in the pathogenesis of vascular and cardiac diseases, Aichara and colleagues fail to mention blood platelets as a potential source of TGFβ. However, this is a very common oversight, and the presence of TGFβ in endothelial cells, vascular smooth muscle cells (VSMCs), myofibroblasts, macrophages and hematopoietic cells, but not blood platelets, has commonly been used to implicate TGFβ in the pathogenesis of cardiovascular diseases, such as coronary artery disease or hypertension. These conditions are associated with the anatomical remodelling of the blood vessels (neointima hyperplasia) and the heart (cardiac hypertrophy) in response to cardiovascular stress factors (hyperglycaemia, mechanical stress, angiotensin II) or in response to local damage to blood vessels in some clinical procedures (angioplasty). All these pathological phenomena are, according to the authors, closely related to the activity of TGFβ, and its activity is particularly pronounced in patients with kidney diseases and metabolic syndrome [55].

Interestingly, all the above-mentioned histological changes observed in the cardiovascular system in response to stressors and leading to cardiovascular disease(s) have a distinctly pronounced haemostatic component with a key contribution of platelets [56,57,58,59]. Therefore, we recommend that blood platelets should be recognized as key sources of TGFβ in any future studies focused on TGFβ-induced pathological remodeling of cardiovascular tissues.

## 8. Platelet TGFβ in Preeclampsia

While preeclamptic women exhibit significantly higher plasma levels of TGFβ in comparison to healthy pregnant women, they also show a lower degree of platelet reactivity. Moreover, women with preeclampsia tend to demonstrate lower numbers of blood platelets [13], which implies that the higher TGFβ concentration in this group may not be a direct result of blood platelet level. Normotensive pregnant and non-pregnant women demonstrate similar levels of TGFβ; however, these levels are significantly lower than in preeclamptic women, which clearly suggests that increased TGFβ may be associated somehow with the pathogenesis of preeclampsia [60]; indeed, women with plasma TGFβ concentrations in the highest quartiles showed the highest risk of preeclampsia [61,62]. This opens the possibility to consider TGFβ plasma levels as a possible diagnostic marker of the risk of preeclampsia: a serious disease of placentation with very little known pathogenesis and very limited diagnostic markers [63]. However, Clausen et al. [64] observed that in women with subsequent preeclampsia, the plasma levels of TGFβ were significantly lower than in control group, suggesting that it is still a matter of debate whether the changes in TGFβ in plasma of preeclamptic women may really reflect the pathological formation of the uteroplacental unit [64].

The very few available published results indicate that although preeclampsia is influenced by TGFβ, the TGFβ produced by the uteroplacental unit exerts a greater influence than that by the platelets. This is a good example of a disease that will raise the vigilance of those who wish to associate any TGFβ-dependent pathology with blood platelets.

## 9. Platelet TGFβ and Liver Diseases

As indicated in mice carrying a megakaryocyte/platelet-specific targeted deletion of the *TGFβ* gene, platelets appear to influence the development of liver fibrosis: they appear to be the primary source of TGFβ stimulating the synthesis of collagen in hepatic stellate cells and favouring their transdifferentiation into myofibroblasts. A very accurate time association was observed between the peak TGFβ concentration in plasma, TGFβ levels in the liver and the activation of blood platelets: all occurred six hours after administration of CCl_4_, the toxicant commonly used in models of liver fibrosis. Moreover, it was observed that transient thrombocytopenia protects the liver against fibrosis [7].

The activation of platelets in the liver, leading to increased local concentrations of TGFβ, and its activation, may result as a consequence of the interaction of blood platelets with extracellular matrix proteins of the liver sinuses exposed by damage to the endothelium [65,66] by oxidative stress [67,68], shear stress [69,70], the action of thrombospondin-1 (TSP-1) [71,72] or proteases [73]. It should be mentioned that the process of liver fibrosis may result not only from the interaction of platelets, and platelet TGFβ, with liver cells including hepatocytes, but also with other types of cells that build the liver, such as residual liver macrophages or hepatic endothelial cells, which show the ability to internalize platelets [74,75].

In the case of liver fibrosis, the role of platelets cannot be absolutely unquestionably presented as leading to harmful effects. Some reports actually indicate that platelets stimulate liver regeneration and have anti-fibrotic properties [76], which may also be related to the action of platelet-derived TGFβ.

## 10. Platelet TGFβ in a Cancer

Ovarian cancer patients, who exhibited a higher rate of metastasis, presented a higher platelet count and higher levels of TGFβ in blood than patients with fewer metastatic foci; this indicates again that higher levels of TGFβ are associated with higher platelet counts. It has also been shown that platelet TGFβ stimulates the epithelial-mesenchymal transition, which could be effectively inhibited by the action of A83-01: an inhibitor of the TGFβ type I receptor [12]. This is a very important observation showing that platelet-derived TGFβ may be a target of pharmacological interventions in cancer.

Platelet TGFβ, complexed and activated by TSP-1, is essential for bone remodelling and the preparation of a premetastatic niche in bones in patients with prostate cancer [77,78]. Hence, platelet TGFβ and TSP-1 appear crucial not only for primary prostate tumour growth, but also for the metastasis to distal tissues, including skeletal tissues. In patients suffering from a prostate cancer, plasma levels of TGFβ may thus predict the chemical recurrence and the risk of bone metastases [79,80,81].

The mechanism related to the platelet transport of TGFβ from the primary prostate tumour cells to the premetastatic niche in the bone is a novel pathway. It confirms some previous reports suggesting that platelets can promote bone metastasis in a mechanism based on osteoblast proliferation, mesenchymal stem cell osteogenesis and the differentiation of osteoclast-like cells [82,83,84,85].

Two different pathways facilitating metastasis appear to be dependent on platelet TGFβ: remodelling of the bone premetastatic niche and the stimulation of epithelial-mesenchymal transition. Bone formation, stimulated by primary tumours, may be effectively inhibited by the depletion of blood platelets, suggesting that blood platelets are the key factors mediating bone metastasis. Of the various proteins known to be involved in bone metabolism (incl. matrix metalloproteinases: MMP-1, MMP-2, MMP-13, receptor activators of nuclear factor κ-B: RANK, receptor activators of nuclear factor κ-B ligand: RANKL, and tissue inhibitor of metalloproteinase, TIMP-2), TGFβ was found to be present in highest amounts in blood platelets taken from animals with a prostate cancer model (inoculation with LNCaP-C4-2 cells); it was described as a protein sequestered and transportedby blood platelets from the primary tumour to the premetastatic niche. In this sense, blood platelets could be regarded as “Trojan horses” for TGFβ. However, this phenomenon is not observed for all types of cancer; for example, it is absent from melanoma.

TGFβ transferred by blood platelets from the primary tumour to the bone is able to stimulate osteoblast differentiation, indicating that it is the blood platelets that determine the pre-metastatic communication between the primary tumour and the target bone tissue [86]. After secretion from the primary tumour, various proteins, including TGFβ, are sequestrated by blood platelets, what protects them against their degradation in a blood [87]. Downregulation of the TGFβ pathway may block communication between the primary tumour and bone microenvironment, and thus may block the formation of the premetastatic niche in skeleton tissues; however, this would result in no alteration in the growth of the primary tumour. It seems that the TGFβ pathway is a promising anti-metastatic molecular target and the concentration of TGFβ in platelets might represent a potential biomarker of cancer aggressiveness [88].

It is worth mentioning that TGFβ derived from blood platelets can also activate the Smad protein and NFκB factor in cancer cells, thus enabling the epithelial-mesenchymal transition and metastasis. Importantly, the specific ablation of TGFβ in platelets seems to have an antimetastatic effect [89].

## 11. Platelet TGFβ in Cerebral Malaria

Blood platelets have been found to be activated by substances secreted by *Plasmodium falciparum*. Circulating resting platelets taken from patients suffering for malaria show decreased expression of activation markers than resting platelets obtained from control subjects; however, following agonist stimulation, the platelets from malaria patients demonstrate greater expression of the markers of activation than non-malaria volunteers, thus indicating a hypersensitive state [90,91,92]. Therefore, in malaria patients, blood platelets may more readily adhere to endothelial cells [93], which also demonstrate a procoagulant phenotype characterised by increased endothelial production of reactive oxygen species, enhanced expression of P-selectin, tissue factor and plasminogen activator inhibitor 1 (PAI-1), and decreased expression of thrombomodulin [94,95]. All these features result in enhanced interactions between blood platelets and endothelial cells, especially at the blood-brain barrier, and greater direct contact between blood platelets and the cerebral endothelium, marked by the significant release of TGFβ from blood platelets. This released platelet TGFβ induces apoptosis of endothelial cells, subsequently leading to the formation of microvascular lesions [96]. Thus, platelet TGFβ may contribute to the pathogenesis of cerebral malaria, and especially to the changes in the permeability of the blood-brain barrier [97] and oedema formation [98].

## 12. Conclusions

The starting point for our deliberations was the statement that amongst the various tested cells in blood, the platelets carry the largest amounts of TGFβ in their granules and are the leading regulator of TGFβ concentration in blood plasma. The mechanism regulating TGFβ secretion from blood platelets must be specific and strictly modulated by appropriate inhibitors and activators, including standard platelet agonists; however, the influence of commonly-recognized platelet agonists on TGFβ secretion remains poorly understood. It certainly appears that shear-stress and classical atherogenic factors, such as hypercholesterolemia, modulate platelet reactivity and significantly influence the secretion of platelet TGFβ. We are also clearly aware that their effects may mutually reinforce each other. In addition, the issue of TGFβ uptake from cells into platelets, and their transport to target tissues, as well as the possibility of attaching TGFβ molecules to the outer platelet membrane, seem to be interesting directions, although yet poorly investigated. However, these phenomena have only been described in single scientific reports and their biological significance remains unclear.

A very important problem is that platelet samples require careful handling when being tested for their ability to secrete TGFβ. Although this problem seems more or less solvable, it may nevertheless account for some of the inconsistency observed in the results, such as the ability of TGFβ to both activate and inhibit platelets or to stimulate or prevent liver fibrosis. Moreover, it should be taken into account that a simple measurement of the concentration of TGFβ in the blood is not sufficient to determine whether TGFβ actually modulates the (patho)physiological process selected for testing; despite this, this procedure is still commonly employed and may entail some inconsistency in the results.

Platelet TGFβ is a crucial factor in heart and coronary vessel remodeling, tumour metastasis, liver fibrosis, permeability of the blood-brain barrier, as well as in tissue regeneration (liver, bone) and the modulation of the immune properties of various types of white blood cells.

On the other hand, it should be noted that blood platelets not only secrete TGFβ, which stimulates some side pathways in target cells, but they are also themselves sensitive to TGFβ and can respond with the use of the appropriate receptors and downstream Smad proteins. Unfortunately, the details and the importance of this signalling pathway in platelets has not been fully investigated and recognized.

This paper presents a rather fragmentary set of results. The issue of platelet TGFβ is poorly investigated, and as such, little is known of the complete biochemical pathways, exact molecular mechanisms or the significance of these processes. However, despite being so poorly explored, this subject is an important one with great clinical impact.

Most of the above conclusions are only based on single reports, and as such, should be regarded as no more than interesting initial findings that require further study

## Data Availability

Not applicable.
